# Digital Health Interventions for Mental Health, Substance Use, and Co-occurring Disorders in the Criminal Justice Population: A Scoping Review

**DOI:** 10.3389/fpsyt.2021.794785

**Published:** 2022-01-20

**Authors:** Rebecca Leach, Stephanie Carreiro, Paige M. Shaffer, Ayorkor Gaba, David Smelson

**Affiliations:** ^1^Division of Medical Toxicology, Department of Emergency Medicine, University of Massachusetts Chan Medical School, Worcester, MA, United States; ^2^Department of Addiction Psychiatry, University of Massachusetts Chan Medical School, Worcester, MA, United States

**Keywords:** digital health, mHealth, telehealth, substance use disorder, mental health, co-occurring disorder, criminal justice

## Abstract

**Background:**

Substance use disorder (SUD), mental health disorders (MHD), and co-occurring mental health and substance use disorders are common among criminal justice populations. Digital health interventions (DHI) represent an opportunity to expand co-occurring disorder treatment for justice involved populations, but efficacy data are lacking.

**Objectives:**

The current scoping review aims to address this gap via following objectives: (1) Describe trends involving DHIs for MHD, SUD, or co-occurring disorders studied in criminal justice settings; and (2) review available evidence for the impact of DHIs on criminal justice-, substance-, and mental health-related outcomes.

**Methods:**

PubMed was searched for relevant articles that met the follow inclusion criteria: (1) focus on criminal justice-involved individuals; (2) description of an intervention focused on SUD, MHD, or co-occurring disorders; and (3) use of DHI. Articles were assessed using standardized data abstraction and quality assessment tools.

**Results:**

Four-hundred unique articles were identified on initial search, and 19 were included in the final review. The most common focus of the intervention was SUDs. The most common modalities were telehealth and computer assisted interventions, with most utilized as an adjunct to treatment as usual. No DHIs used wearable devices, and one included justice involved youth. Feasibility and acceptability were high, and the studies that measured substance and mental health-related outcomes reported equivocal or positive results. No studies focused on long-term justice-related outcomes.

**Conclusions:**

Literature on DHIs for criminal justice involved populations diagnosed with SUD, MHD and co-occurring disorders is limited, and largely focuses on telehealth or eHealth, with less data on mHealth approaches. Future research should focus on the inclusion of diverse populations and include objective monitoring tools.

## Introduction

Substance use disorders (SUDs) are a public health crisis in the United States (US), with more than 90 thousand overdose deaths in 2020 (1). Additionally, 88,000 people die annually from alcohol-related causes—the 3rd leading preventable cause of death in the US (2). Providers struggle to help clients with SUDs access and remain engaged in treatment and support services. The National Survey on Drug Use and Health (NSDUH) estimates that 19.3 million U.S. people have SUD. Of those, 49.2% have co-occurring substance use and mental health disorders (3). Regrettably, in 2019, NSDUH reported the majority of U.S. adults with co-occurring disorders did not receive either mental health or specialty SUD treatment in the past year, and many are involved in the criminal justice system. Furthermore, recent estimates indicate that over 70% of incarcerated persons have co-occurring disorders and often cycle in and out of treatment and criminal justice systems due to untreated co-occurring disorders, and drug-related offenses (4–8).

Although there are effective treatments available across criminal justice settings, and a high demand for behavioral health services, relatively few justice-involved individuals with SUD receive treatment. The Bureau of Justice Statistics reported that among the incarcerated population who met the criteria for drug dependence or abuse, only 28% of individuals in prisons and 22% of individuals in jail had participated in a drug treatment program since admission (9). Data looking broadly at justice involved individuals found that only 38% received any type of services for SUD or MHD within their lifetimes, and of which only 7% received services for co-occurring disorders (10). Several treatment barriers have been identified, including limited staff training knowledge, stigma, high staff turnover, lack of resources, workforce shortages impacting facilities in rural areas, and fragmented reentry services (11–13).

Digital health, or the use of information/communication technology to facilitate healthcare (14), could be a cost-effective solution that addresses some of these unique challenges. Digital health interventions (DHIs) encompass many facets of technology including: (1) telehealth or telemedicine, which is used by health care providers to deliver real-time treatment over distance through videoconferencing or audio technology; (2) mHealth, otherwise known as mobile health, or the delivery of care that supports health objectives via mobile or wireless devices, which includes, but not limited to, mobile phones, mobile applications, patient monitoring devices, and wearable devices (15); and (3) eHealth, which is a broad term used to describe health services and information delivered or enhanced through the internet and related technologies such as web-based or computer assisted platforms (16). Digital health has shown promise as a vehicle to deliver healthcare to the general public with SUD, MHD, and co-occurring disorders. A systematic review evaluating the current usability and impact DHIs for SUD reported high acceptability of this technology among the SUD population with the majority of studies showing positive results with respect to efficacy (17). Another systematic review evaluated the available digital health technologies for people with a serious mental illness and found that digital health technology was used for a wide range of applications including knowledge gain, clinical use, and intervention with overall results showing high acceptability, feasibility, and efficacy. Furthermore, it was determined that digital health technologies for serious mental illness could be useful when incorporated into long term treatment (18).

To the best of our knowledge, only one systematic review examined DHIs for criminal justice populations (12), which compared telepsychology services delivered through videoconferencing vs. in-person services delivered to incarcerated individuals with SUD. Telepsychology was found to be at least comparable to in-person visits, however the authors argue a need for more evidence due to the overwhelming lack of a control group in most studies and small sample sizes. Several gaps remain in the current literature. First, there are no reviews that evaluate the literature for the various types of DHI in criminal justice populations with SUD or MHD. With the increasing rate of technology development, various forms of DHI should be explored together to compare efficacy and identify areas to focus future efforts. Additionally, because of the high prevalence of co-occurring disorders in the criminal justice population and the unique needs of this population, it is useful to evaluate the existing literature on this diagnostic category as well. The current review aims to address this gap by evaluating the literature on DHIs for MHD, SUD, or co-occurring disorders in the criminal justice population with the following objectives: (1) Describe trends in clinician type, disease focus, target population, intervention type and outcomes studied, and (2) review available evidence for the impact on justice-, substance-, and mental health-related outcomes.

## Materials and Methods

### Search Strategy

The protocol and search methodology were developed in accordance with support from a medical research librarian, and was conducted in accordance with Preferred Reporting Items for Systematic reviews and Meta-Analyses Extension for Scoping Reviews (PRISMA-ScR) Guidelines (19). A search for relevant articles containing keywords related to criminal justice involvement, substance use disorder, mental health, co-occurring disorders, and digital health interventions was conducted using PubMed. Articles published through April 29, 2020 were included in the search. The full search string is outlined in Appendix 1.

### Eligibility

Articles screened for the following inclusion criteria: (1) focus on juvenile and/or adult populations involvement in the criminal justice system (including populations on probation, in prison, on parole, or re-entry into the community after being released from prison); (2) description of an intervention focused on SUD, mental health, OR co-occurring disorders; (3) use of mHealth/ telehealth or e-Health (including usual care vs. to mHealth/telehealth OR usual care with addition of mHealth); and (4) original research, including but not limited to randomized control trials, pre-post studies with no control, feasibility/acceptability studies, and qualitative studies. For the purpose of this review, telephone only interventions did not qualify as an mHealth/telehealth intervention, an the term DHI refers to any described intervention that met inclusion criteria 2 and 3. Articles were excluded if they were: (1) not in English language; or (2) a systematic review, letter to the editor, protocol, or case report.

### Study Selection

A single reviewer (RL) manually screened the initial list of titles and abstracts of identified articles and removed those that obviously screened out based on inclusion/exclusion criteria. Full texts were obtained for all screened in articles by a single reviewer (RL). For any questions with eligibility, a second reviewer (SC) reviewed the articles independently, and any discrepancies were discussed until consensus was reached.

Extracted information included: year published, percent female, age range, study location, study type; disease focus, study design, the population type, clinician type, details and description of the intervention, purpose of the study, and the key findings.

### Quality Assessment

The National Heart, Lung, and Blood Institute's study quality assessment tools were used to assess quality of quantitative studies; the controlled intervention studies, pre-post studies with no control, or a case-control study tool was used depending on study type (20). Studies were graded as “good” if 70% or more of the questions were answered with “yes”, fair if 30–60% of the questions were answered with “yes”, and poor if 30% or less of the questions were answered “yes” or there was a fatal flaw identified. For qualitative studies, the Critical Appraisal Skills Programme (CASP) tool for qualitative research was used to assess quality (21). The CASP tool did not include a rating scale however we followed the same grading scale described for quantitative studies above (i.e., rating as good, fair, or poor). Each article was evaluated by two authors (RL and SC) and any discrepancies were discussed until consensus was reached. The ratings obtained were strictly used to provide an overall quality assessment of the articles included in this review. Inclusion or exclusion of an article in this review was not determined by the quality assessment.

## Results

### Study Selection Process

**Figure 1:**
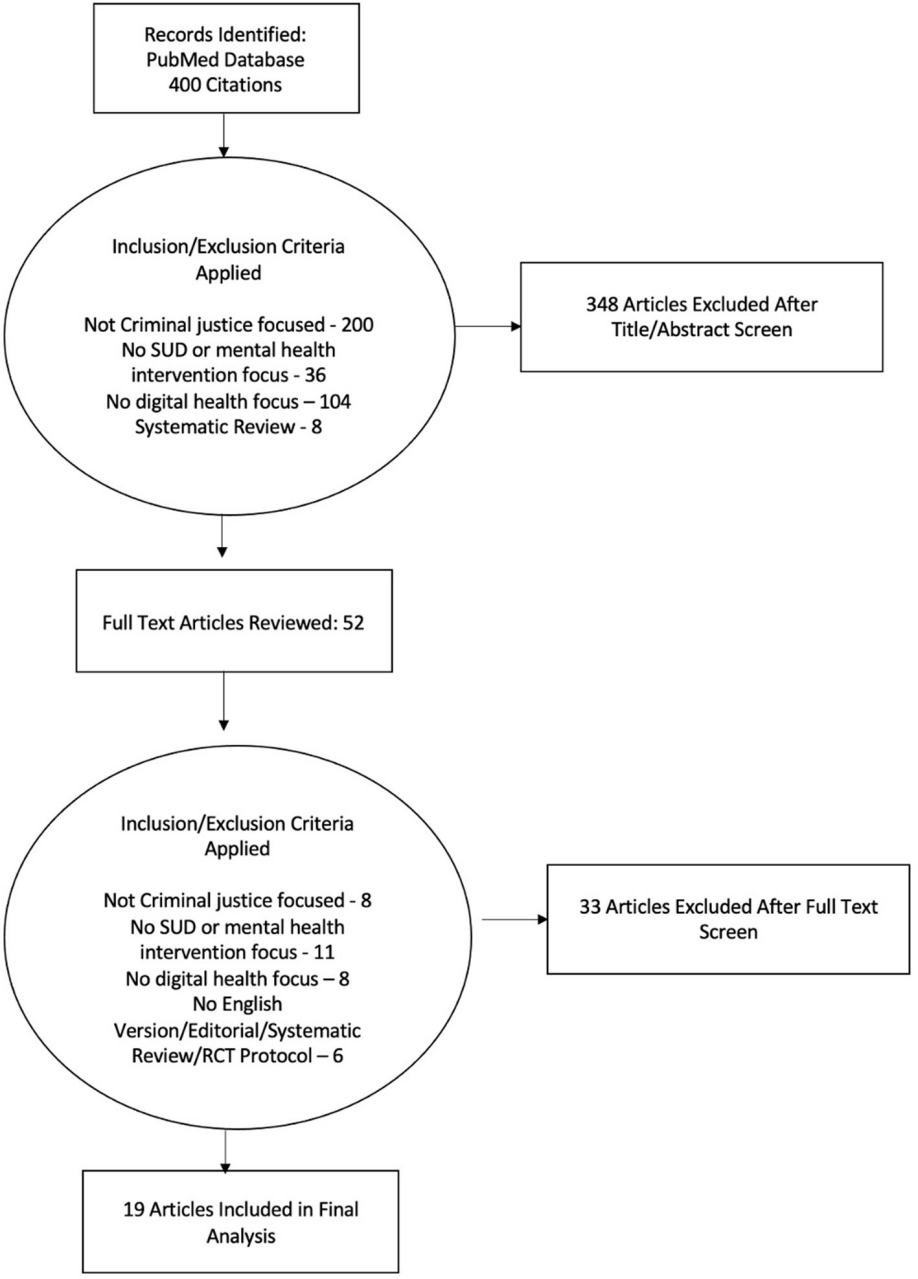
Outline of study selection process.

The results for the study selection process are outlined in Figure 1. Four hundred articles were identified in the initial search, which were reviewed by title and abstract and excluded if (1) there was no criminal justice population focus, (2) no mental health or SUD focus, or (3) no eHealth/telehealth/mHealth focus. Full texts were reviewed for the remaining 52 articles. Thirty-three of these articles were deemed ineligible based on the above criteria, leaving a total of 19 eligible articles (13, 22–39). Of note, five of the articles included in the final set related to the same parent study (22–25, 39).

### General Study Characteristics

**Figure 2:**
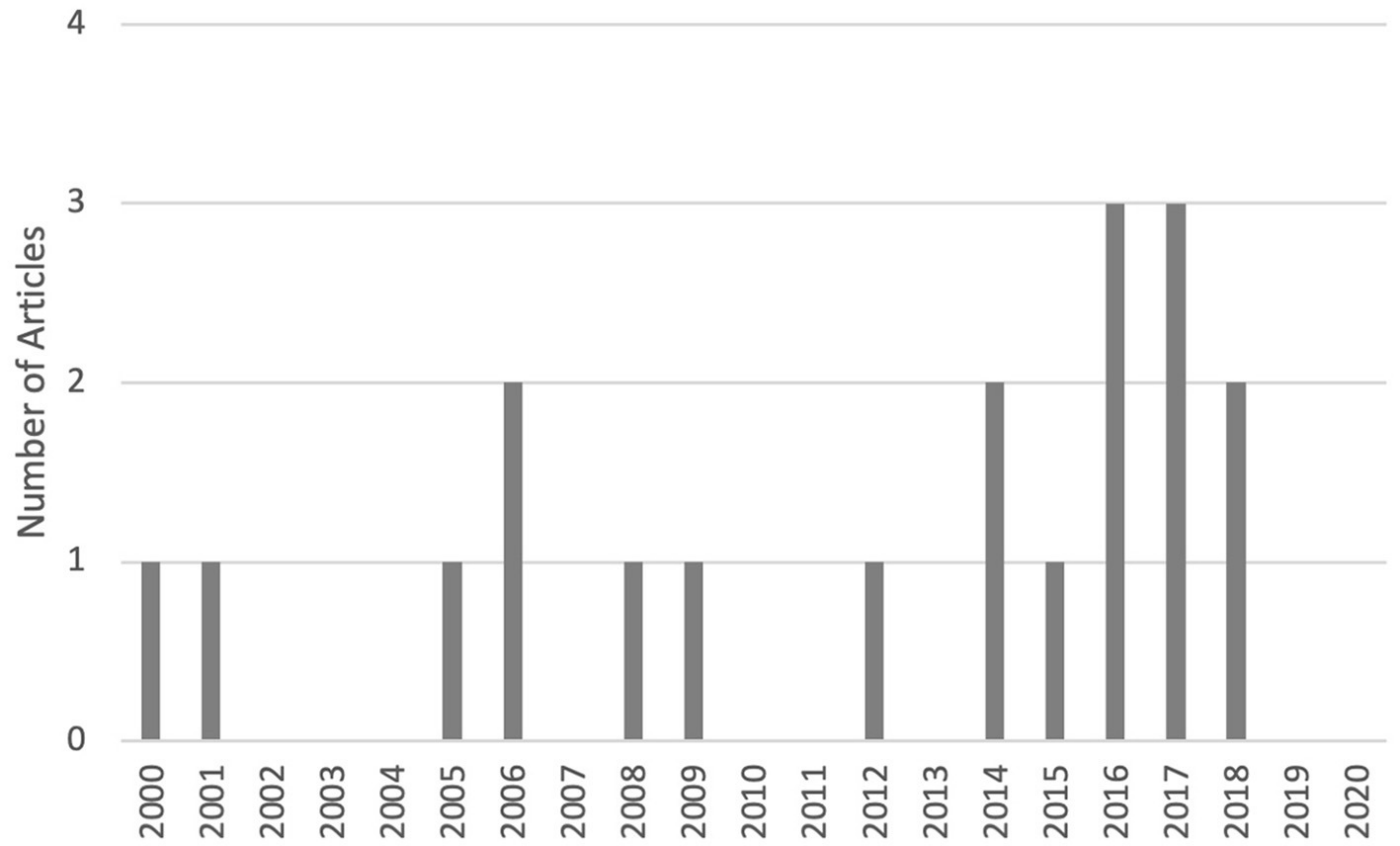
Temporal distribution of reviewed articles.

**Figure 3:**
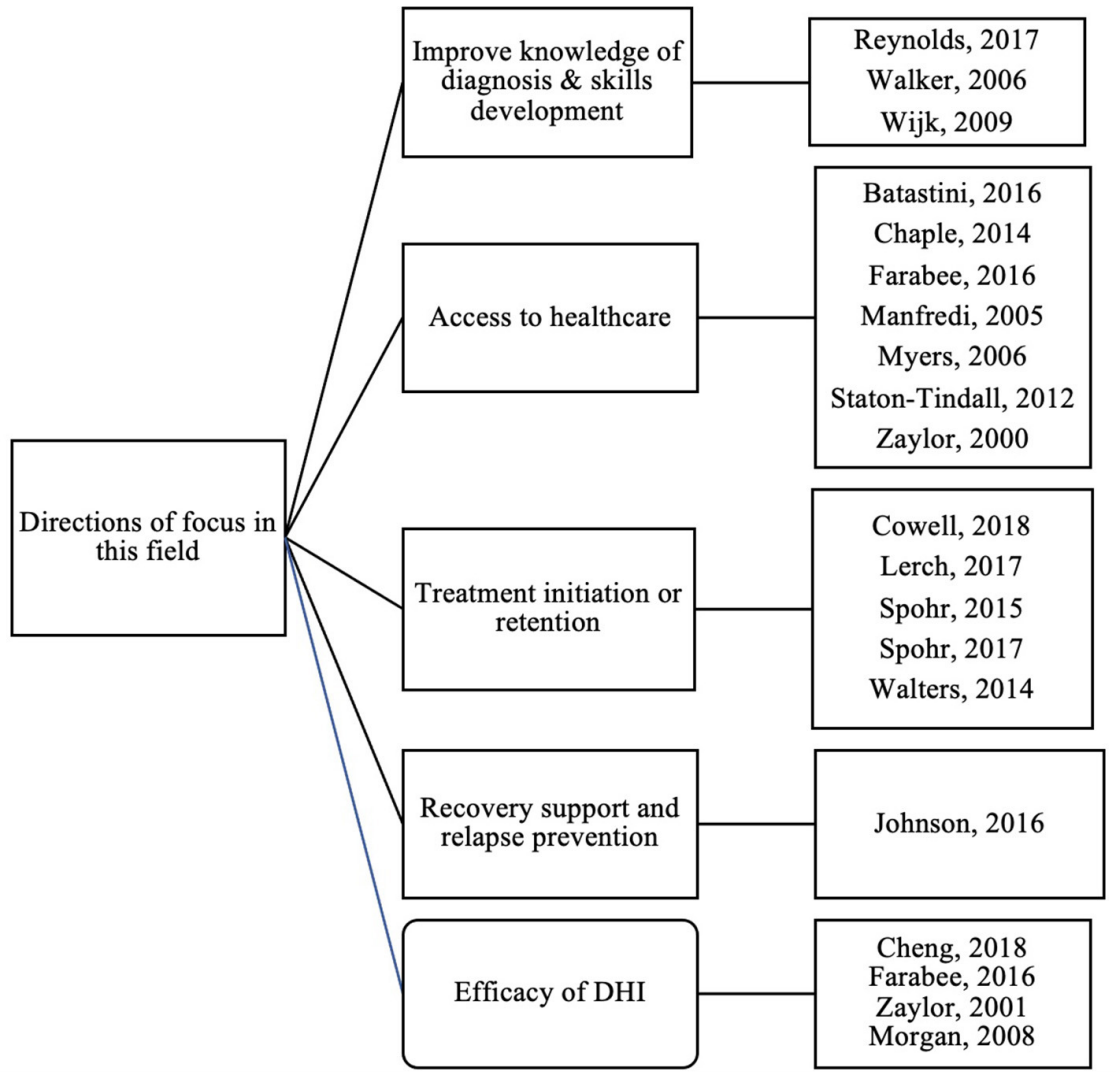
Directions of focus for included studies (*Of note, included studies may fall into* >*1 category*).

An overview of the eligible articles is included in Table 1. The temporal distribution of the articles over the study period is shown in Figure 2; of note no included articles were published in 2019 or 2020. Only one article focused on juvenile offenders (33), while the other 18 articles focused solely on adult populations. In terms of study location, the majority of articles were conducted in the United States (15 out of 19), while one was from England (27), one from Scotland (35), one from Sweden (36), and one from China (28). Major directions of study include diagnostics/skills development, access to healthcare, treatment initiation/retention, recovery support and relapse prevention, and efficacy of DHIs (Figure 3). With regards to study type, five articles described randomized controlled trials (RCTs) (23–25, 29, 30), one described a non-randomized controlled trial (32), ten described pilot or feasibility/acceptability studies (13, 26–28, 31, 33–36, 39), one described a cost-effectiveness analysis of an RCT (22), and two articles described observational studies (37, 38). Of the eligible articles, digital health modalities used included telehealth or videoconferencing (*N* = 9) (13, 28, 29, 31–34, 37, 38), computerized (or computer assisted) interventions (*N* = 8) (22–25, 30, 35, 36, 39), mobile phone based (*N* = 1) (26), and serious gaming (e.g., game designed for a purpose other than strict entertainment, *N* = 1) (27). Of note, no studies used a wearable device, other sensor devices, or any version of physiologic monitoring.

**Table 1:** Overview of eligible articles.

**Record ID**	**Disease focus**	**Study design**	**Population type**	***N***	**% female**	**Age range (years)**	**Intervention name**	**Clinician type**	**Duration**	**Purpose**	**Key findings**
**Computerized/computer assisted interventions**											
**Chaple** **(****30****)**	SUD	RCT	Prisoners	494	30.4%	Mean age 36.6	Therapeutic Education System (TES)	Self- administered	12 weeks	Evaluate the feasibility of a computerized intervention (TES) in a prison by measuring inmate participation, satisfaction, and skills acquisition	•TES had high rates of module completion •Both experimental and control groups showed significant improvement coping strategies over time, with no significant difference between groups
**Cowell** **(****22****)**	SUD	CEA of RCT	Probationers	316	NR	Adults, range NR	MAPIT	Self-administered	3–4 week	Assess the cost-effectiveness of a computerized motivational intervention (MAPIT) to motivational interviewing +treatment as usual	•MAPIT cost less per person on probation than motivational interviewing for motivating treatment initiation
**Lerch** **(****23****)**	SUD	RCT	Probationers	316	NR	Adults, range NR	MAPIT	Self-administered	3–4 weeks	Compare the effectiveness of a computerized motivational intervention (MAPIT) vs. an in-person motivational interviewing vs. standard probation intake, measured by treatment initiation and substance use	•MAPIT significantly improved treatment initiation at short-term follow up •No significant impact on substance use
**Spohr** **(****24****)**	SUD	RCT	Probationers	113	NR	18 - 63	MAPIT	Self-administered	3–4 weeks	Evaluate the reliability and predictive validity of a brief survey about individual's reasons for wanting to complete probation	•Motivation by freedom, legal, relationships, and time chosen associated with fewer days of substance use •Motivation by relationships and shame associated with higher treatment attendance •Motivation by financial reasons associated with fewer days of treatment
**Spohr** **(****25****)**	SUD	RCT	Probationers	76	NR	19–62	MAPIT	Self-administered	3–4 weeks	Determine if choosing to receive text or email reminders about their probation and treatment goals would increase achieving early treatment initiation and probation tasks	•Those who chose to receive electronic reminders also tended to choose more goals, had less days of substance use, and had more days of treatment compared to those did not
**Walker** **(****35****)**	MHD	Qualitative Pilot	Forensic mental health prisoners	10	20%	22–46	NR	Self-administered	4–5 sessions, 1 h each session	Evaluate the use and acceptance of a computer-delivered relapse prevention plan in the attempt to improve patients' knowledge of their disease, psychosis	•Forensic patients indicated high usability and acceptability of the CD-ROM program •Forensic patients were able to develop and follow their relapse prevention plan
**Walters** **(****39****)**	SUD	Pilot	Probationers	21	NR	Adults, range NR	MAPIT	Self-administered	3–4 weeks	Describe the development and overview of a computerized motivational intervention (MAPIT) program and to report initial testing results	•Initial testing reported high positivity toward the MAPIT program, especially the accuracy and usefulness
**Wijk** **(****36****)**	MHD	Pilot	Mentally disordered offenders (MDOs)	21	12.5%	Adults, range NR	Reactions on Display (RoD)	Sessions led by an MD and resident	1 session	Develop and pilot a computer simulation system (RoD) used for the rehabilitation of MDOs and as a tool for staff to learn more about their patients' risk factors	•RoD was accepted by patients and staff in terms of design, realism, engagement, and enjoyability •Further research should include clinical outcomes.
**Mobile phone based**											
**Johnson** **(****26****)**	SUD	Pilot	Outpatient drug court participants	30	13%	Adults, range NR	A-CHESS	Self-administered	4-months	Determine if drug court participants would utilize a smartphone app (A-CHESS) to aid in recovery	•Participants used A-CHESS on an average of 62% of days while enrolled in the study •Social networking tool was the most used feature
**Serious game**											
**Reynolds** **(****27****)**	MHD	Feasibility/ acceptability	Forensic mental health prisoners	228	0%	Adults, range NR	StreetWise	Self-administered	1 session	Determine feasibility and acceptability of a serious game to improve discharge results	•Serious games were acceptable and feasible •Further work and development of this technology needs to add more complexity
**Telemedicine**											
**Batastini et al**. **(****12**, **13****)**	MHD	Pilot	Prisoners	49	0%	Adults, range NR	Coping Skills Group (CSG)	Master's Level MHD provider	6-weeks	Implementing group telepsychology intervention to isolated inmates	•Telepsychiatry intervention was not associated with meaningful improvements in psychological functioning •Telepsychiatry was less favorable than in person •No significant differences of psychological functioning and criminal thinking between groups
**Cheng** **(****28****)**	MHD	Pilot	Prisoners	335	0%	21–64	NR	Psychiatrist	Up to 4 sessions	Compare psychiatric care delivered via teleconsultations or in-person to persons in custody	•Significant improvement in the Chinese-General Health Questionnaire (C-GHQ-12) score post intervention in teleconsultation group •High satisfaction and favorable response to teleconsultation.
**Farabee** **(****29****)**	MHD	RCT	Parolees	104	26%	Mean age 38.1	NR	Psychiatrist	6-months	Evaluate the effectiveness of telepsychiatry delivered to parolees with psychiatric disorders	•High satisfaction with telepsychiatry •Comparable results for psychological functioning and medication adherence •Decline in therapeutic alliance in the videoconferencing group
**Manfredi** **(****31****)**	MHD	Pilot feasibility	Prisoners	15	13%	Mean age 21	NR	Psychiatrist	NR	Determine if telepsychiatry consultation is a feasible method to increase mental health access to rural jails	•High acceptability from patients, jail staff, psychiatrist, and social worker
**Morgan** **(****32****)**	MHD	Non-randomized controlled trial	Prisoners	186	0%	Mean age 31.8	NR	Psychologist and psychiatrist	1 session	Examine therapeutic alliance and inmates' mood, satisfaction, and perception toward tele-mental health services	•No significant difference between groups regarding working alliance, satisfaction, or mood.
**Myers** **(****33****)**	MHD	Feasibility	Juvenile prisoners	115	24%	13–19	NR	Psychiatrist	1–9 visits (avg 2.4 visits)	Feasibility of telepsychiatry service implemented in a juvenile correction facility	•Results supported satisfaction with telepsychiatry and suggests that this modality can be used to deliver psychopathology successfully to juvenile prisoners
**Staton-Tindall** **(****34****)**	SUD (alcohol)	Feasibility	Community supervision	75	9.2%	19–57	Motivational enhancement therapy (MET)	Therapist	4 sessions over 12 weeks	Describe a new telemedicine program that delivers an alcohol intervention and to determine its feasibility among a group of at-risk alcohol users	•MET is a feasible and acceptable program for the delivery of alcohol abuse services to at-risk probationers or parolees
**Zaylor** **(****38****)**	MHD	Observational	Prisoners	70	11%	Mean age 29	NR	Psychiatrist	NR	Determine acceptability among patients and jail staff of a telemedicine project implemented in a jail after 1 year	•Patients received the telepsychiatry services well •Jail staff reported positive experiences
**Zaylor** **(****37****)**	MHD	Observational	Prisoners	45	9%	NR	NR	Psychiatrist	2 months	Determine if telepsychiatry is effective from the perspective of both the patient and the provider	•Psychiatric distress decreased over time •Psychiatrists reported patient improvement over time

*NR, Not reported; SUD, Substance Use Disorder; MHD, Mental Health Disorder; RCT, Randomized Controlled Trial; IOP, Intensive Outpatient Program; CEA, Cost-effectiveness Analysis*.

### Clinician Type

All interventions were either self-administered (i.e. mobile app, computer- based automated interventions or serious games, *N* = 9) (22–27, 30, 35, 39) or conducted by clinical staff (i.e. psychiatrists, psychologists, or masters level mental health providers *N* = 10) (13, 28, 29, 31–34, 36–38). No articles described DHIs that involved peer support personnel/recovery coaches (13, 28, 29).

### Disease Focus (SUD vs. MH vs. Co-occurring Disorders)

Out of the 19 articles included in this study, eight articles focused on DHI for SUD only (some general SUD, and some focused on DHI for specific SUDs such as stimulant use disorder or alcohol use disorder) (22–26, 30, 34, 39), and eleven articles focused on mental health only (13, 27–29, 31–33, 35–38). Among the eight SUD focused studies, five studies focused on a computerized intervention (22–24, 30, 39), one focused on a computerized intervention with a text and email add-on (25), one study focused on a smartphone app (26), and one study utilized telemedicine (34). Among the eleven MH focused studies, eight studies focused on telehealth video conferencing with a psychiatrist (13, 28, 29, 31–33, 37, 38), two focused on a computer intervention (35, 36), and one study focused on delivering a serious game intervention to help plan for patient discharge (27).

### Study Design, Interventions and Inclusion of Treatment and Usual

As the optimal role of DHIs in the treatment paradigm is yet to be seen, included studies used various study designs and implementation methods to investigate the DHI efficacy. Some aimed to compare DHI to treatment as usual, and others evaluated it as an adjunct. Eight articles evaluated DHI alone (27, 31, 33–38). Two articles evaluated DHI + treatment as usual with no comparison group (26, 39). The remaining 9 articles described the DHI +/- treatment as usual compared to a treatment as usual only group (13, 22–25, 28–30, 32). Four articles evaluated their DHI + treatment as usual in comparison to treatment as usual (22–25). Of note, all four of these articles described the MAPIT DHI, a computer-based intervention to motivate participants and promote engagement in treatment, in addition to treatment as usual (22–25). The five remaining articles used DHI as a stand-alone treatment and compared that to treatment as usual; four were telemedicine-based interventions (13, 28, 29, 32) and one was a computerized intervention (30).

### Population Type and Outcomes

Among the studies included in this sample, a variety of criminal justice settings and sub-populations were included to determine efficacy of DHI with heterogeneous outcome measures. We present outcome results based on population type as the measures evaluated were most similar in these domains.

Seven articles examined DHI in incarcerated populations (13, 28, 30–32, 37, 38). The DHI's implemented for the incarcerated populations included one computer assisted intervention (30) and six telemedicine intervention (13, 28, 31, 32, 37, 38). The majority reported positive results with high acceptability with patients/staff (28, 31, 38), and improvement in psychiatric symptoms (37) and improved coping strategies (30). One study reported equivocal results, specifically no difference between the DHI telepsychiatry evaluation when compared to traditional face to face evaluation (32), which may be taken as a positive result to demonstrate non-inferiority of the DHI. A single study reported less favorable outcomes when using a DHI (telepsychiatry) compared to face-to-face evaluations (13). However, the authors suggested the lack of group differences were more likely related to problems in treatment delivery rather than the delivery method itself, and should not be used to discount the use of telepsychology as a viable treatment delivery option.

Seven articles examined DHI in community supervision populations; five of these studies examined clients on probation (22–25, 39), one study focused on a parolee population (29), and one study included probationers or parolees (34). The DHIs implemented in these studies included five computerized/computer assisted interventions (22–25, 39) and two telemedicine interventions (29, 34). As in the incarcerated population studies, the results were largely positive with high acceptability and/or perceived usefulness (29, 34, 39) and improvement in clinical outcomes, including increased treatment engagement (23, 25), decreased substance use (24, 25). One study demonstrated a cost benefit for a computer based intervention (22). One study noted that despite high satisfaction, and comparable clinical results when compared to treatment as usual, the DHI (telepsychiatry) group showed decreased therapeutic alliance over time (29).

Three articles examined the use of DHI in clinical (e.g., forensic mental health) settings within the justice system, two including computer assisted interventions (35, 36) and one testing a serious game DHI (27). All were pilot or feasibilities studies and all reported high usability and acceptability among forensic patients, but also acknowledged that further work was needed to design effective interventions in this space.

One article focused on the use of DHI in an alternative to incarceration strategy (Drug Treatment Court), specifically the use of a smartphone app to enhance drug court outcomes (26). Findings indicated that most drug court participants in this sample made regular use of the recovery support app, and in particular used a messaging feature to engage in peer group discussions.

One article focused on the use of DHI for justice involved juveniles, specifically to determine feasibility of telepsychiatry services for individuals within a juvenile correction facility (33). Results showed satisfaction with the intervention, but there was a concern about privacy. Overall, the telepsychiatry were found to be an acceptable modality to deliver services to justice involved juveniles.

### Perceived Bias and Quality Ratings of Included Studies

Of the nineteen articles included articles, 17 were assessed for quality using NHLBI quality assessment scales: six articles were controlled intervention studies (13, 22, 23, 29, 30, 32), one was a case-control study (28), four were pre-post studies with no control group (24–26, 37), and six were observational studies (31, 33, 34, 36, 38, 39). Two articles were assessed for quality using the CASP scale, (27, 35)..

Of the nineteen articles assessed, 26% were rated as good or valuable, 74% were rated as fair, and none of the articles were rated as poor. There were no clear differences in reported outcomes in the good vs. fair groups of articles. Many of the articles rated as fair largely were penalized in the rating scales for small sample sizes and/or lack of blinding. Of note, all of the controlled interventions used an intent-to-treat analysis, which improved the overall robustness of the results.

## Discussion

In this scoping review of DHIs, several themes arose, including: a heavy focus on SUDs (as opposed to MHDs or co-occurring disorders); integrations of DHIs with treatment as usual as opposed to use as stand-alone interventions; and focus on relatively basic DHI technology such as telehealth or computer assisted interventions. Studies using mobile phones/apps were uncommon, and no studies using wearable or other non-invasive sensors were found. Feasibility and acceptability (in studies where addressed) were generally high.

The articles reviewed did not address which stage of justice involvement would benefit the most from DHIs. There was some evidence of benefit in all levels- incarcerated individuals, those on community supervision and those in clinical settings within the justice system. However, the populations and outcomes studied varied widely, so comparisons are difficult to make. No studies specifically addressed the impact of DHIs on recidivism or other long term justice-related outcomes.

Telepsychological approaches, particularly those involving videoconferencing, have the potential to foster safer, more intensive, and arguably more humane interactions with treatment providers than what is typically afforded to administratively segregated inmates. People in rural prisons where access to mental health, SUD, or co-occurring disorder interventions is especially limited provide a particular opportunity for DHIs. However, at least one study suggested lower levels of perceived therapeutic alliance for telepsychiatry (29), and one suggested that in-person treatment was sometimes preferred (13). While the authors caution that these results may be related to the execution rather than the technology, they raise important concerns about the unintended consequences of using a digital format to deliver even standard interventions. While privacy and trust concerns (and their impact on utilization and efficacy) are always central considerations for DHIs, they are arguably even more important in a vulnerable population that may be hesitant to engage at baseline.

The influence of individual characteristics, such as sex/gender, race, ethnicity, disability, and age on the uptake and efficacy of DHIs is important to consider so that DHIs can be tailored for maximal benefit. Many studies were predominantly male, presumably due to the sex-based division in the criminal justice setting. This introduces bias and limits the generalizability of findings. Additionally, only one article focused on juvenile offenders, which ironically is a population expected to be more accepting of (and comfortable with) DHIs given the current “connected” culture (40, 41). Future work is needed to understand how DHIs will need to be tailored to individuals based on sex/gender, race, ethnicity, disability and age.

The timing of interventions with respect to the stage of involvement with the criminal justice system is an important consideration for both the content of the DHI and the metric by which we evaluate their success. Some studies discuss prisoners receiving intervention from services and/or telehealth services while incarceration, however there is little description of whether these services were terminated or continued upon release. This begs the question of whether continued utilization would provide an added benefit, and whether the intervention type needs to change with the stage of justice involvement. For example, transition back into the community is a critical time to educate and motivate clients, so a DHI aims at re-entry populations may work toward developing goals that will help address substance use and other risk behaviors. And additional consideration is the jail vs. prison setting, and what implications the distinction has on optimal DHI usage.

The gaps in the literature also provide some important insight. For example, the lack of articles from the last 2 years may be indicative of fading interest in the topic or reflective of the difficulty inherent in research in the criminal justice system in general. However, with some DHIs (such as telemedicine) being more mainstream, data on use and efficacy may be captured in program evaluations that are not being published in the medical literature.

Interestingly, no DHIs described in these included articles used wearable devices, mobile phone sensors, or other continuous passive data collection tools. This has been previously reported in DHIs that target SUD (17). Concerns regarding privacy and reluctance of justice involved individuals to be monitored may drive researchers and clinicians away from these technologies. However, prior literature supports that notion that well deployed opt-in interventions can be highly acceptable in traditionally stigmatized populations (17, 42). Wearable technology has the potential for sensors to detect substance use and behavioral states that place individuals at high risk for return to drug use (i.e., stress, drug craving) (17, 42, 43). Data from wearable devices can also be integrated with other sensors (e.g., GPS from mobile phones) and contextual data to drive predictive analytics, which identify periods of risk and prime opportunities for just-in-time adaptive interventions. Given the high risk for relapse and recidivism in this population, this represents a potential missed opportunity and area for future work.

The overall quality of articles included in the review was fair to good. A substantial portion of the articles rated “fair” due to some challenges inherent in digital health research. For example, large samples sizes can be challenging due to cost of technology, and the time sensitive nature of mHealth research- waiting too long to complete a study may result in a lapse in technology. Technology based studies may struggle with blinding (due to the physical presence of the technology), which naturally introduces bias and decreases quality ratings (based on standard quality scales). Overall, larger studies and more randomized controlled trials are needed to increase the robustness of this body of literature.

Many of the DHIs evaluated were intended to be self-administered adjuncts to routine care. Some facilitated a provider interaction (for example a counseling session with a psychiatrist or other licensed provider). However, none utilized the DHI as a way to engage individuals with peer support professionals, which may represent a missed opportunity. The use of peer support personnel, or individuals with lived experience and formal training, has become an increasingly popular care model in the criminal justice settings. Engaging peer support personnel adds a human component to the DHI without requiring time from already stretched clinicians. The common choice to add DHI to treatment as usual compared to DHI alone speaks to the utility of DHIs in general as an adjunct (but not necessarily a replacement for) excellent clinical care.

Implementation challenges unique to DHIs are important to consider when assessing feasibility and potential impact in the justice involved population, and may be particularly problematic in the transition or re-entry period. Equipment availably and internet access may be an issue in DHIs, specifically those that require a mobile phone or personal computer. Digital health literacy may also effect uptake, and was not addressed in the included studies.

Much work is left to do with regard to the design, implementation, and effectiveness of DHIs in criminal justice settings. Based on the currently available literature, suggestions for future research include: (1) Understanding DHI use and efficacy in diverse populations including women, juvenile offenders, and ethnically diverse samples to tailor and personalize approaches; (2) Evaluating the impact of DHI long term outcomes such as recidivism, return to substance use, and engagement in treatment; (3) Addition of continuous, objective monitoring tools (e.g., wearable sensors) and predictive analytics to deliver just-in-time interventions; (4) Engagement of peer support professionals in DHI administration; and (5) Exploration of DHI characteristics that work best as stand-alone interventions compared to adjuncts to treatment as usual.

Obtaining a complete picture of the DHI research landscape is challenging due to some inherent limitations. Terminology associated with DHIs often includes multiple interchangeable expressions to refer to a single concept. Despite our extensive search terms we may have missed some articles that used alternative keywords, for example. Furthermore, industry-based studies are not typically included in the medical literature, due to concerns over intellectual property and proprietary information. The commercial “gray literature” on DHI is difficult to find and would have been missed by our search strategy. The published literature may overestimate effectiveness due to positive publication bias. Finally, we limited our search to only English language articles, which would cause us to miss key articles published in other languages; these would be particularly important to consider in the context of cultural factors that would influence outcomes.

Literature on DHIs in SUD, MHD and co-occurring disorders in the criminal justice population is limited despite the population prevalence and need for additional treatment options; and largely focuses on telehealth and eHealth, with limited data on mHealth approaches. Future research on DHIs in this population should focus on the inclusion of diverse populations, understanding the impact of DHIs at various stages in the justice system, and the inclusion of mHealth and objective monitoring tools.

## Data Availability Statement

The original search strategy used to identify articles reported in this review are included in the article/Supplementary Material, further inquiries can be directed to the corresponding author.

## Author Contributions

SC and DS conceptualized the study. RL and SC performed the original literature search, abstracted data from the included study articles, and discussed all conflicts in the data abstraction process. SC, RL, PS, AG, and DS synthesized and analyzed the data. All authors contributed significantly to the compilation of results and the synthesis of the manuscript.

## Funding

This work was generously supported by Substance Abuse and Mental Health Service Administration (SAMHSA) Center for Substance Abuse Treatment grant awarded to the Massachusetts Department of Public Health Bureau of Substance Addiction Services (Award Number TI081717, Sub-awardee: DS), and National Institutes of Health/National Institute on Drug Abuse (R44 DA046151, PI: SC).

## Conflict of Interest

The authors declare that the research was conducted in the absence of any commercial or financial relationships that could be construed as a potential conflict of interest.

## Publisher's Note

All claims expressed in this article are solely those of the authors and do not necessarily represent those of their affiliated organizations, or those of the publisher, the editors and the reviewers. Any product that may be evaluated in this article, or claim that may be made by its manufacturer, is not guaranteed or endorsed by the publisher.
