# Supplementary material for: Digital Health Interventions for Mental Health, Substance Use, and Co-occurring Disorders in the Criminal Justice Population: A Scoping Review
Source: Front Psychiatry. 2022 Jan 20;12:794785. doi: 10.3389/fpsyt.2021.794785 (PMC8811209; doi:10.3389/fpsyt.2021.794785)
Supplement: Supplementary file 1 [file Data_Sheet_1.docx]

Supplementary Material

**Digital Health Interventions for Mental Health, Substance Use and Co-Occurring Disorders in the Criminal Justice Population: A Scoping Review**

**Appendix 1**

("Forensic Psychology"[Mesh] OR forensic psychology[TIAB] OR "Forensic Psychiatry"[Mesh] OR forensic psychiatry[TIAB] OR correctional psychology[TIAB] OR correctional psychiatry[TIAB] OR forensic patient*[TIAB] OR forensic client*[TIAB] OR "Prisoners"[Mesh] OR prison*[TIAB] OR jail*[TIAB] OR detention[TIAB] OR "Criminals"[Mesh] OR “Criminal Law"[Mesh] OR criminal*[TIAB] OR offender*[TIAB] OR parole*[TIAB] OR probation[TIAB] OR acquitee*[TIAB] OR re-entry[TIAB] OR reintegration[TIAB] OR court[TIAB] OR courts[TIAB])

AND

("Community Mental Health Services"[Mesh] OR community mental health service*[TIAB] OR assertive community treatment[TIAB] OR community-based treatment*[TIAB] OR community forensic service* OR "Secondary Prevention"[Mesh] OR secondary prevention*[TIAB] OR secondary disease prevention*[TIAB] OR relapse prevention*[TIAB] OR "Psychiatric Rehabilitation"[Mesh] OR psychiatric rehabilitation[TIAB] OR mental health rehabilitation[TIAB] OR psychosocial rehabilitation[TIAB] OR "Social Work, Psychiatric"[Mesh] OR social work[TIAB] OR social service*[TIAB] OR recovery[TIAB] OR "Community Integration"[Mesh] OR community integration[TIAB] OR "Continuity of Patient Care"[Mesh] OR continuity of patient care[TIAB] OR continuum of care[TIAB] OR care continuity[TIAB] OR care continuum[TIAB] OR patient care continuity[TIAB] OR transitional rehabilitation housing[TIAB] OR transitional housing[TIAB] OR "Home Care Services"[Mesh] OR home care[TIAB] OR domiciliary care[TIAB] OR substance abuse service*[TIAB] or substance abuse treatment[TIAB] OR "Mental Disorders/rehabilitation"[Mesh] OR "Mental Disorders/therapy"[Mesh] OR "Substance-Related Disorders/rehabilitation"[Mesh] OR "Substance-Related Disorders/therapy"[Mesh] OR "Behavior, Addictive/rehabilitation"[Mesh] OR "Behavior, Addictive/therapy"[Mesh] OR rehabilitat*[TIAB] OR therap*[TIAB] OR "Health Services Accessibility"[Mesh] OR "health services accessibility"[TIAB] OR "availability of health services"[TIAB] OR "health services availability"[TIAB] OR "access to health care"[TIAB] OR "accessibility of health services"[TIAB] OR "program accessibility"[TIAB] OR "Health Services Needs and Demand"[Mesh] OR "needs and demand"[TIAB] OR target population*[TIAB] OR health services need*[TIAB] OR health services demand*[TIAB])

AND

(“Telecommunications”[Mesh] OR telecommunication* OR “Telemedicine”[Mesh] OR telemed*[TIAB] OR teleHealth[TIAB] OR eHealth[TIAB] OR mHealth[TIAB] OR “mobile health”[TIAB] OR “connected health”[TIAB] OR “digital health”[TIAB] OR “Electronic Mail”[Mesh] OR email*[TIAB] OR “Telephone”[Mesh] OR telephone*[TIAB] OR phone*[TIAB] OR “Cell Phone”[Mesh] OR “Smartphone”[Mesh] OR smartphone*[TIAB] OR iphone*[TIAB] OR mobile[TIAB] OR "Mobile Applications"[Mesh] OR mobile app*[TIAB] OR electronic app*[TIAB] OR portable software app*[TIAB] OR wearable[TIAB] OR “Text Messaging”[Mesh] OR text[TIAB] OR texts[TIAB] OR texting[TIAB] OR “Remote Consultation”[Mesh] OR “remote consultation”[TIAB] OR “remote consultations”[TIAB] OR “remote consult”[TIAB] OR teleconsult*[TIAB] OR “Distance Counseling”[Mesh] OR “distance counseling”[TIAB] OR “e-therapy”[TIAB] OR “e-counseling”[TIAB] OR “Therapy, Computer assisted”[Mesh] OR computer*[TIAB] OR “home monitor”[TIAB] OR “home monitors” [TIAB] OR “home monitoring” [TIAB] OR “remote monitor” [TIAB] OR “remote monitors” [TIAB] OR “remote monitoring” [TIAB] OR telemonitor*[TIAB] OR telepsychiatry[TIAB] OR “virtual visit” [TIAB] OR “virtual visits” [TIAB])

AND

("Diagnosis, Dual (Psychiatry)"[Mesh] OR dual diagnosis[TIAB] OR dual diagnoses[TIAB] OR co-occuring[TIAB] OR "Behavior, Addictive"[Mesh] OR addictive behavior*[TIAB] OR "Substance-Related Disorders"[Mesh] OR "substance-related disorder"[TIAB] OR "substance-related disorders"[TIAB] OR "drug abuse"[TIAB] OR "drug dependence"[TIAB] OR "drug addiction"[TIAB] OR "substance use disorder"[TIAB] OR "substance use disorders"[TIAB] OR "drug use disorder"[TIAB] OR "drug use disorders"[TIAB] OR "substance abuse"[TIAB] OR "substance abuses"[TIAB] OR "substance dependence"[TIAB] OR "substance dependences"[TIAB] OR "drug habituation"[TIAB] OR "Addiction Medicine"[Mesh] OR "addition medicine"[TIAB] OR "addiction psychiatry"[TIAB] OR "Analgesics, Opioid"[Mesh] OR opioid*[TIAB] OR "Heroin Dependence"[Mesh] OR "Heroin"[Mesh] OR heroin[TIAB] OR "Alcoholism"[Mesh] OR "Alcohol Drinking"[Mesh] OR "Alcoholic Beverages"[Mesh] OR "Alcohol-Related Disorders"[Mesh] OR "Ethanol"[Mesh] OR ethanol[TIAB] OR alcohol[TIAB] OR "Alcohol-Induced Disorders"[Mesh] OR alcohol-induced disorder*[TIAB] OR "Cocaine Smoking"[Mesh] OR "Crack Cocaine"[Mesh] OR

"Cocaine-Related Disorders"[Mesh] OR "Cocaine"[Mesh] OR cocaine[TIAB] OR crack[TIAB] OR "Benzodiazepines"[Mesh] OR benzodiazepine*[TIAB] OR "Marijuana Use"[Mesh] OR "Marijuana Abuse"[Mesh] OR "Marijuana Smoking"[Mesh] OR "Cannabis"[Mesh] OR cannabis[TIAB] OR marijuana[TIAB] OR "Mental Disorders"[Mesh] OR mental disorder*[TIAB] OR mental illness*[TIAB] OR psychiatric diagnos*[TIAB] OR psychiatric disease*[TIAB] OR psychiatric disorder*[TIAB] OR psychiatric illness*[TIAB] OR "Mental Health Services"[Mesh] OR mental health service*[TIAB] OR mental hygiene service*[TIAB] OR "Mentally Disabled Persons"[Mesh] OR mentally disabled[TIAB] OR mental illness*[TIAB] OR psychiatric disorder*[TIAB] OR "Mood Disorders"[Mesh] OR mood disorder*[TIAB] OR affective disorder*[TIAB] OR "Personality Disorders"[Mesh] OR personality disorder*[TIAB] OR inadequate personality[TIAB] OR impulse-ridden personality[TIAB])
